# Avoidance of Water Bodies by Gaur at Parsa National Park (PNP): Real Ecology of the Species or Methodological Shortfalls?

**DOI:** 10.1002/ece3.71953

**Published:** 2025-08-04

**Authors:** Biplov Sharma

**Affiliations:** ^1^ Tribhuvan University Kathmandu Nepal

## Abstract

Gaur ( *Bos gaurus* ) is one of the largest and threatened ungulates in Nepal with limited ecological researches. To address the gap, Bhattarai et al. have published the article in 2025. However, the paper has many scientific limitations that could mislead the investment made in conservation of species. Their limitations include the issues related to referring gaur as preferred prey despite existing literature referring them as the prey species often avoided by tiger, failure to randomize the sampling necessary to acquire the answer to their research questions and selection of site for placement of camera traps using convenience sampling. Furthermore, despite using the occupancy model, they have failed to account detection probability. Issues of model checking and consideration of credible interval in the response curve have been ignored. When credible interval is included, the response of the species to the given variables becomes questionable. Details of the comments have been explained in the full text.

## Graphical abstract

In 2025, an article by Bhattarai et al. ignored the basic ecology of Gaur. There are methodological and analytical shortcomings, which are not mentioned in the journal. The adequacy of the model has not been tested. There are issues with interpretation of data.

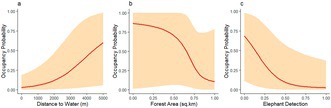

Large herbivores make significant ecological impacts through their influence on the redistribution of organic matter, the indirect suppression of smaller organisms, and the maintenance of spatiotemporal heterogeneity (Pringle et al. 2023). Gaur ( *Bos gaurus* ), an animal with a body size of nearly 600–1000 kg (Ahrestani 2018; Ashokkumar et al. 2011), is distributed in a few countries of Asia, and Nepal is one of its range countries (Duckworth et al. 2016). Gaur are experiencing a wide array of threats and are now listed as a vulnerable species on the IUCN Red List of Threatened Species (Duckworth et al. 2016). Despite the ecological significance of the species, research on Gaur is particularly limited in Nepal (Bhattarai et al. 2025; Dhakal et al. 2025; Poudel et al. 2024). Recently, a group of authors published a research article (Bhattarai et al. 2025) that can lay a foundation for future studies. However, the research paper has many scientific limitations that could misguide conservation efforts. The key points of concern are as follows:

1. Issues with the hypothesis:

The hypotheses of the research relate to habitat use by Gaur in relation to tiger and elephant presence, forest cover, water sources, and human activity (Bhattarai et al. 2025). Among these five hypotheses, the one related to tiger presence neglects already established facts. For instance, Gaur are reported to be significantly avoided by tigers (Hayward et al. 2012). Although tigers occasionally hunt larger prey, they mostly prefer prey of a size equivalent to their own. However, the authors have hypothesized the decrease in occupancy of Gaur in areas with higher tiger due to predation risk.

2 Issues with the methodology:

When a set of hypotheses is defined for a study, the research design must justify those hypotheses. Particularly, if the aim was to test hypotheses related to distance variables, a systematic survey design should have been employed, with cameras placed at varying distances from the variables of interest. For instance, in the study (Bhattarai et al. 2025) if the authors were interested in exploring the habitat use pattern of Gaur with respect to water, they should have placed camera traps taking distance from water sources into account.

3 Issues with camera trap placement:
  - The study area map provided by the authors shows 20 grids with 67 camera traps deployed across them. However, the number of camera traps per grid varied from one to four, without adequate explanation for such variation in sampling frequency.
  - Authors have used the convex hull of the camera traps to show the adequacy of the camera trap effort. This is a poor indicator for estimating the spatial coverage of camera trap coverage as it calculates the area based on the distance of the camera traps at the extreme end.
  - The minimum distance between camera trap locations is reported as at least 1 km (Bhattarai et al. 2025). However, no information is provided about how distances were measured or how local‐level site selection for camera trap placement was conducted. Furthermore, they have ignored the spatial autocorrelation between the sampling points on the results of their model.
  - Based on the map shown in the article, most camera trap locations are distributed in a linear fashion, with overrepresentation in some areas and underrepresentation in others. Furthermore, the northern side of the park was avoided, citing hilly and difficult terrain, despite past studies showing that Gaur use hilly terrains up to 2800 m elevation (Ashokkumar et al. 2011).
4 Grid treatment inconsistency:

On grid‐based survey, sampling area and sampling efforts are defined on the basis of area of total grid surveyed and each grid survey as a sampling unit. Observation made within the area of sampling unit has to be used as the grid level evidence. Given that authors have used varying number of grids in each grid, they should account for this variation in effort in their occupancy model. Wildlife detection is a tricky issue and depends on a variety of factors including sampling efforts, experience of the field researchers, and so on. For all sampling events, the efforts measured in terms of time, experience, and number of cameras should be equal or they should be modeled using appropriate tools. However, after conducting the survey based on grids, the authors treated each camera trap location as a separate event. If each point was intended to be treated independently, establishing grids was unnecessary.

5 Ignoring heterogeneity of detection:

In occupancy studies, heterogeneity of detection is often modeled (Karavarsamis and Huggins 2019). However, the authors did not use any site‐level or habitat‐level covariates to model detection. Additionally, they used Inverse Weighted Distance (IWD) to map detection probability, even though IWD is sensitive to cluster sampling (Benmoshe 2025).

6 Lack of clarity on elephant detection variable:

Although the authors used elephant detection as a covariate, they did not explain how this variable was calculated.

7 No model checking:

After building the model, accuracy and adequacy need to be tested; however, the authors do not mention any model‐checking procedures.

8 Credible intervals hidden in the figures:

Distance to water (m), forest area (km^2^), and elephant detection were found to be significantly associated with Gaur habitat use probability (Bhattarai et al. 2025). Although upper and lower credible intervals were mentioned in the table, they were omitted from the graphical representation. The figure (Figure 1) shows very wide credible intervals, indicating that generalizing these results to the entire Gaur population of Parsa National Park is risky.

**FIGURE 1:**
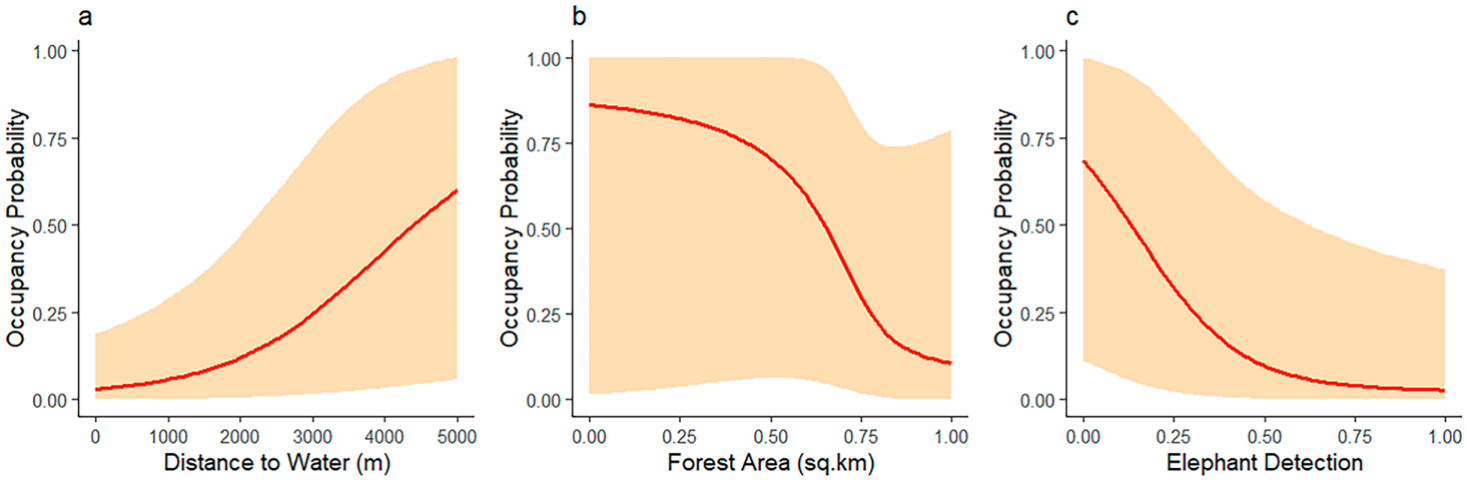
Response of Gaur to distance from water, forest area (km^2^), and elephant detection, keeping all variables constant. The figure has been prepared based on the dataset and code provided by the authors (Sharma 2025), with slight modifications to the code to change color and add credible intervals.

9 Issues with interpretation:
  - A significant positive relationship between Gaur habitat use and distance from water bodies has been interpreted as reduced competition and predation risk near water sources. This is probably a typographical error. The correct phrasing would be “potentially to reduce competition and predation risk.”
  - While interpreting the response to forest area and elephant detection, the authors ignored the credible intervals.
  - They reported that Gaur has a restricted distribution in Chure. However, since their sampling was restricted to the plain area, they lack sufficient evidence to make that claim.
  - As mentioned earlier, the sampling strategy is inappropriate to make claims about the response to distance from water bodies. Gradient analysis such as the change in the space use by the species with respect to the distance should employ a systematic sampling framework and place their study unit at different distances from their variable of interest, that is, water in the case of this paper. However, the authors have placed camera traps in a convenient manner without due consideration of water holes or water sources in the process.
  - No evidence was provided regarding better forage availability at forest edges or open grasslands and its relation to distance from water bodies. Given their massive body size, it is highly unlikely that Gaur can completely avoid water bodies, especially during the dry season. It is surprising that the authors overlooked this common ecological understanding.

As journal articles are the foundation on which evidence‐based conservation is planned, these issues need to be adequately addressed before using the publication for further use.

## Author Contributions

**Biplov Sharma:** conceptualization (lead), formal analysis (lead), writing – original draft (lead), writing – review and editing (lead).

## Conflicts of Interest

The author declares no conflicts of interest.

## Data Availability

As this is Letter to the Editor, there are no separate data associated with this letter. The data shared by original authors have been used.
